# Label-Free 3D Ag Nanoflower-Based Electrochemical Immunosensor for the Detection of *Escherichia coli* O157:H7 Pathogens

**DOI:** 10.1186/s11671-016-1711-3

**Published:** 2016-11-17

**Authors:** He Huang, Minghuan Liu, Xiangsheng Wang, Wenjie Zhang, Da-Peng Yang, Lianhua Cui, Xiansong Wang

**Affiliations:** 1 Department of Plastic and Reconstructive Surgery, Shanghai Key Laboratory of Tissue Engineering, National Tissue Engineering Center of China, Shanghai 9th People’s Hospital, Shanghai Jiao Tong University School of Medicine, Shanghai, People’s Republic of China; 2 College of Chemical Engineering & Materials Science, Quanzhou Normal University, Quanzhou, China; 3 School of Medicine, Qingdao University, Qingdao, China

**Keywords:** 3D Ag nanoflowers, Electrochemical immunosensor, *E. coli*, Bovine serum albumin

## Abstract

It is highly desirable to develop a rapid and simple method to detect pathogens. Combining nanomaterials with electrochemical techniques is an efficient way for pathogen detection. Herein, a novel 3D Ag nanoflower was prepared via a biomineralization method by using bovine serum albumin (BSA) as a template. It was adopted as a sensing interface to construct an electrochemical bacteria immunosensor for the rapid detection of foodborne pathogens *Escherichia coli* (*E. coli*) O157:H7. Bacterial antibody was immobilized onto the surface of Ag nanoflowers through covalent conjugation. Electrochemical impedance spectroscopy (EIS) was used to detect and validate the resistance changes, where [Fe(CN)_6_]^3−/4−^ acted as the redox probe. A linear relation between *R*_et_ and *E. coli* concentration was obtained in the *E. coli* concentration range of 3.0 × 10^2^–3.0 × 10^8^ cfu mL^−1^. The as-prepared biosensor gave rise to an obvious response to *E. coli* but had no distinct response to *Cronobacter sakazakii*, methicillin-resistant *Staphylococcus aureus* (MRSA), *Staphylococcus albus*, *Lactobacillus easei*, and *Shigella flexneri*, revealing a high selectivity for the detection of the pathogens down to 100 cfu mL^−1^ in a short time. We believe that this BSA-conjugated 3D Ag nanoflowers could be used as a powerful interface material with good conductivity and biocompatibility for improving pathogen detection and treatment in the field of medicine, environment, and food safety.

**Electronic supplementary material:**

The online version of this article (doi:10.1186/s11671-016-1711-3) contains supplementary material, which is available to authorized users.

## Background

The risk being infected by pathogenic bacteria in food and drinking water is one of the major concerns on human health. Detection and identification of harmful organisms, for example, *Salmonella typhimurium* and *Escherichia coli* O157:H7, in a cost-effective, rapid, and selective manner remains a challenging task. Conventional methods including bacterial culture, counts of colonies, and use of the polymerase chain reaction and immunological techniques such as ELISA are time-consuming, labor intensive, and often produce nonspecific results. Thus, these methods no longer meet the requirements of pathogenic bacteria diagnosis in food, in the clinic and the general environment [1]. Therefore, it is important to develop effective techniques for disease prevention, for medical diagnosis, and to ensure food safety.

The introduction of nanotechnology has provided important new insights into the problems involved in pathogen detection and identification [1]. Up to now, a great number of nanomaterials including noble metal nanoparticles, quantum dots, and carbon nanomaterials as well as metal oxide nanoparticles have been actively explored for the detection of pathogenic bacteria [2–4]. Taking advantage of their unusual attributes, such as optical, electrical, magnetic, and acoustic properties, various biosensors including surface-enhanced Raman scattering (SERS), fluorescence, and surface plasmon resonance, as well as electrochemical (amperometric, impedance, and luminescence) biosensors based on nanomaterials and recognition elements, have been developed [5–9]. Of all the biosensors, electrochemical impedance spectroscopy (EIS) biosensors have emerged as extremely useful tools for pathogen detection by antigen-antibody interactions on nanomaterial-modified electrode surfaces [3, 10]. EIS works by detecting alterations in an electrochemical system over a wide range of sine wave frequencies. The output signal is electrical current, whose intensity is a function of frequency [9], with a Nyquist impedance graph commonly used to determine the electron transfer resistance, *R*_et_. When bacteria affix to an electrode surface, they reduce the output current and thus increase the impedance of the interface. *R*_et_ increases with increasing bacterial cell concentration [11]. Nanomaterials on the electrode increase the number of biomolecules that become immobilized (due to the large surface-volume ratio) and then amplify the biomolecular recognition events, resulting in a greatly enhanced signal. By constructing a recognition interface on the electrode that is biocompatible (using biofunctionalized nanomaterials), label-free electrochemical cytosensing can be achieved [2]. On the one hand, the interface material should possess good conductivity, which could accelerate the electron transfer; on the other hand, it also should afford a highly stable and biocompatible matrix, which is fit for the attachment and growth of cells [12].

In an earlier study, we generated noble metal microspheres (Au, Ag, and Pt) to investigate their potential electrochemical sensing applications [13–16]. The preparation method was based on a green pathway, where bovine serum albumin (BSA) was used as a template and stabilizing agent. In the present study, we utilized a modified method to obtain BSA-conjugated Ag nanoflower architectures with attractive features (widely open porosity, large surface area, intrinsic conductivity, and unique platform for functionalization) to act as an electrochemical sensing interface for *E. coli* detection. The first aim of the present study was to develop an electrochemical impedance system capable of detecting various types of bacteria, with BSA-conjugated Ag nanoflowers employed as the working electrode. The second aim was to evaluate the effectiveness of Ag@BSA nanoflowers compared with previously reported nanomaterials.

## Methods

### Chemicals and Materials

Ascorbic acid and AgNO_3_ were sourced from Sinopharm Chemical Reagent Co. (Shanghai, China). Bovine serum albumin (68 kDa) was bought from Xiamen Sanland Chemicals Company Limited. The anti-*E. coli* O157 antibody was purchased from Abcam (Hong Kong) Ltd. A [Fe(CN)_6_]^3−/4−^ solution containing 10 mM K_3_Fe(CN)_6_, 10 mM K_4_Fe(CN)_6_, and 0.1 M KCl (the supporting electrolyte) was used as the redox probe. Phosphate-buffered solution (PBS, 10 mM, pH 7.4) containing 14 mM KH_2_PO_4_, 87 mM Na_2_HPO_4_, 2.7 mM KCl, and 137 mM NaCl was employed to dilute the anti-*E. coli* antibody and also as the washing solution. A Millipore Milli-Q system was used to produce ultrapure water. Analytical grade reagents were used in all experiments; it was deemed unnecessary to purify them further.

### Synthesis of 3D Ag Nanoflowers

The synthesis of Ag nanoflowers was as previously described [17]. Briefly, a 10 mL BSA solution (5 mg/mL) and a 10 mL AgNO_3_ solution (10 mM) were added to a 50-mL beaker and magnetically stirred for 10 min at room temperature and then placed in a water bath for 5 min at 25 °C. Subsequently, 50 mg of ascorbic acid was rapidly added and the mixture maintained at 55 °C for 30 min, before being allowed to cool to room temperature. The Ag nanoflowers were harvested and washed three times with water and then three times with ethanol. Finally, the sample was stored in a refrigerator (4 °C) for subsequent use as required.

### Experimental Apparatus and Measurement Equipment

A field emission scanning electron microscope (FESEM, ZEISS ULTRA 55) and a transmission electron microscopy (TEM, JEOL 2011) were used to examine the morphology of Ag@BSA nanoflowers. A CHI 660D electrochemical workstation (Shanghai CH Instruments Co., China) was used to carry out EIS and differential pulse voltammetric (DPV) experiments. The electrochemical cell consisted of three compartments, namely, a modified Au electrode served as the working electrode, a platinum wire served as the auxiliary electrode, and the reference electrode was a saturated calomel electrode (SCE). Electrochemical measurements were carried out in sterile PBS containing K_3_Fe(CN)_6_/K_4_Fe(CN)_6_ (10 mM, 1:1) and 0.1 M KCl (10 mM, pH 7.4). A frequency range of 10^−2^–10^5^ Hz was used to record the impedance spectra (signal amplitude 5 mV). Nyquist plots were generated using ZSimpWin software (ver. 3.10). DPV experiments were performed with a CHI 660B electrochemical workstation which utilized a conventional three-electrode system (vide supra). CV measurements were carried out in PBS containing 5 mM K_3_Fe(CN)_6_ and 0.1 M KCl (10 mM, pH 7.4). A scan rate of 100 mV s^−1^ was employed over a −0.2-V and +0.8-V range. Fourier transform infrared (FTIR) spectrophotometer measurements were made using a Bruker EQUINOX 55 FTIR spectrometer (range 4000–400 cm^−1^). X-ray diffraction measurements were performed using a Bruker AXS D8 instrument at 40 kV and 40 mA with Cu-Kα radiation (*λ* = 1.5406 A).

### Bacteria Culture

All bacterial strains (including *E. coli*, *Cronobacter sakazakii*, and MRSA) used in this study were purchased from the Institute of Microbiology, Chinese Academy of Sciences. Prior to use, they were incubated twice at 37 °C for 24 h in tryptic soy broth. The initial concentrations were determined using plate counting and serial dilution techniques. Experiments involving the subculture of pathogenic bacteria, their maintenance, and various treatments were performed in a level II biosafety cabinet.

### MTT Assays

Human skin fibroblast cells were obtained from the cell bank of the Chinese Academy of Sciences and cultured in RPMI 1640 medium supplemented with 10% FBS. Cells were incubated for 2–3 days at 37 °C in a humidified chamber containing 5% CO_2_. Subsequently, 100 μL volumes containing 1 × 10^4^ cells were added to well plates and incubated for 24 or 48 h, respectively. The cell medium was then replenished and various concentrations of Ag@BSA nanoflowers added. The control groups consisted of human skin fibroblast (HSF) cells alone. After removal of the medium containing the matrix, 20 μL of a solution of 5 mg/mL of MTT was added and incubation was initiated for 4 h. Then, 150 μL of DMSO was added and the solution agitated for 15 min.

### Assembly and Recognition of Bacteria Biosensors Based on Ag@BSA Nanoflowers

**Fig. 1:**
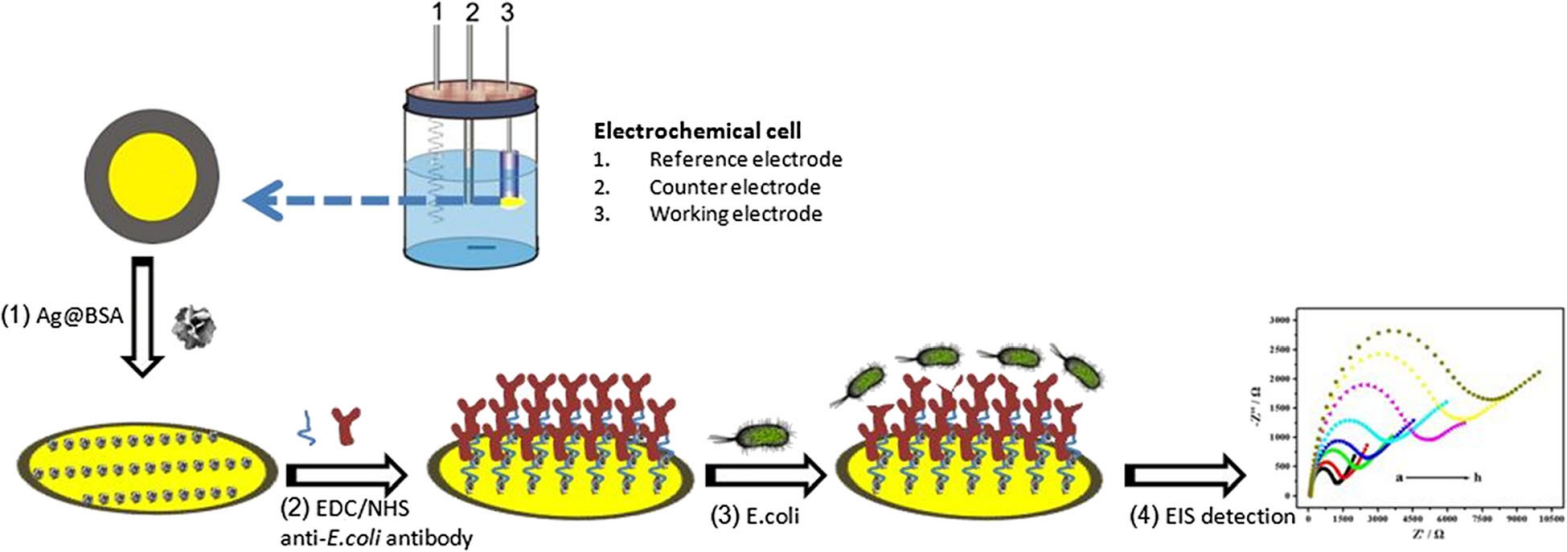
Schematic illustration of immunosensor fabrication. *1* The coating of Ag@BSA nanoflowers on the surface of the Au electrode. *2* Surface activation by using EDC/NHS and antibody binding onto Ag@BSA nanoflowers. *3* Pathogen capturing based on the prepared immunosensor. *4* EIS measurement

The bacterial immunosensor basis of Ag@BSA nanoflowers is shown schematically in Fig. 1. Antibodies are anchored to BSA by an amide coupling technique. A polished Au electrode (2.0-mm diameter) was washed ultrasonically in water and ethanol and cleaned using a mixture of piranha solution (3:1, 98% H_2_SO_4_/30% H_2_O_2_) for 3 min and rinsed with deionized (DI) water and ethanol. After drying the electrode under a stream of gaseous nitrogen, a 3 μL of Ag@BSA nanoflower solution (1.5 mg mL^−1^) was gently applied to the Au electrode surface. The unbound Ag nanoflowers were washed out using PBS (pH 7.4) (Fig. 1 (1)). To enable anti-*E. coli* antibody attachment, 2.5 μL of a 25% glutaraldehyde solution was added together with the Ag@BSA nanoflowers to form a cross-linked organic layer. The excess aldehyde groups on the Ag@BSA were chemically deleted by the application of NaBH_4_. Then, the carboxylic acid groups on the Ag@BSA molecules were activated by reaction with a mixture of EDC/NHS. Excessive EDC/NHS was removed by centrifugation, and intermediate *O*-acylisorurea compounds formed amide bonds with the amine groups of the antibodies (Fig. 1 (2)). Next, a 5 μL suspension of *E. coli* at different cell concentrations was applied to the surface of the modified Au electrode and incubated at 37 °C for 2 h to achieve cell immobilization, followed by the antibody-antigen interaction (Fig. 1 (3)). The unbound bacteria were gently removed by rinsing with sterile PBS at pH 7.4. Finally, the electrochemical responses of the [Fe(CN)_6_]^3−/4−^ probe were recorded using EIS. The electron transfer resistance (*R*_et_) is a measure of the number of bacteria interacting with the immunosensor (Fig. 1 (4)).

## Results and Discussion

### Characterization of Immunosensors Based on Ag@BSA Nanoflowers

**Fig. 2:**
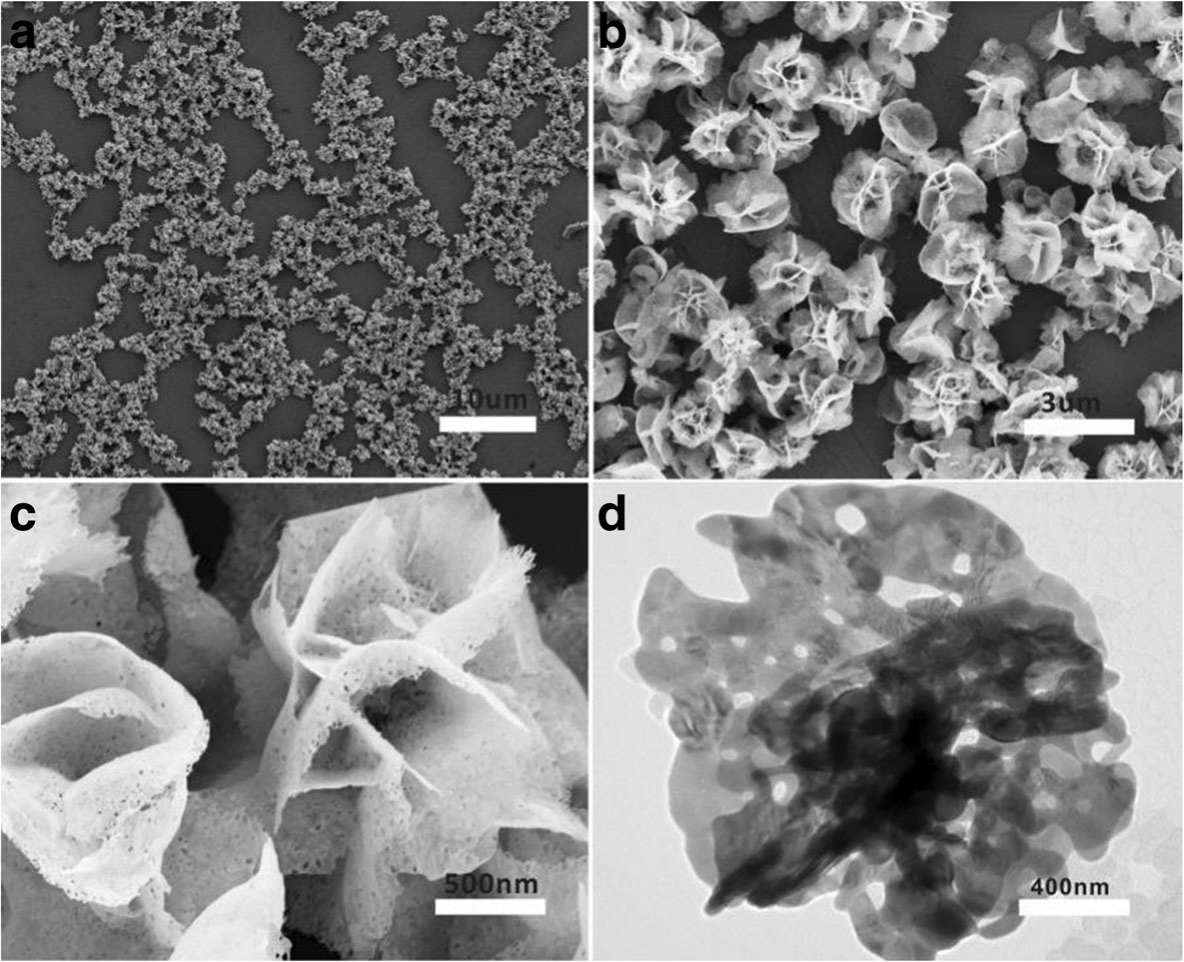
FESEM (**a**–**c**, from lower magnification to higher magnification) and TEM image (**d**) of a single 3D Ag@BSA nanoflower

**Fig. 3:**
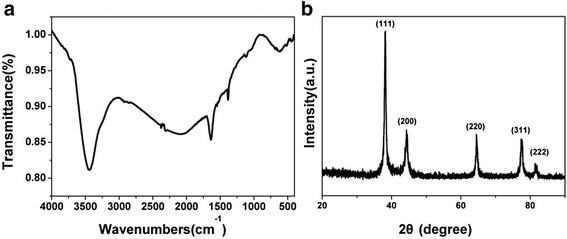
**a** FTIR spectrum and **b** XRD pattern of 3D Ag nanoflowers

It is of great importance to develop high-performance nanomaterials with strong electrical conductivity and excellent biocompatibility for their use in the manufacture of electrochemical biosensors. Herein, we chose Ag@BSA nanoflowers as sensing interfaces to develop an effective method to detect bacteria. The advantages of nanoflowers are that they can be synthesized in an environmentally friendly manner, are very stable, and have a highly specific surface area as well as excellent biocompatibility. As shown in Fig. 2a, the resulting products had nearly spherical shapes with an average size of ca. 2 μm in diameter and good dispersivity. It can be clearly found that the as-prepared products had many rough surfaces and multi-layer structures. EDX result showed that the products were exclusively composed of Ag (Additional file 1: Figure S1). A higher magnification SEM image indicated that these rough surfaces had 3D flower-like structures, called Ag nanoflowers (Fig. 2b). The 3D nanoplatforms will help to strengthen local topographic interactions between bacterial surfaces and Ag@BSA sensing layer. The petals on the Ag nanoflower surfaces stretched to different directions, which had a thickness of ca. 10 nm. Interestingly, the enlarged SEM image (Fig. 2c) indicated that the petals were porous structures with different size distributions. It is worth noting that there is no Ag nanoflower formation without BSA addition (Additional file 2: Figure S2). Typical TEM image of a Ag nanoflower in Fig. 2d provided us further insight into its structure; one can see that each piece of petal indeed was made up of some small interconnected Ag nanoplates, forming the 3D multi-layer porous structures. The contrast between the dark inner and the bright outer parts revealed that the petals grew from the center of the Ag nanoflower. In addition, a thin layer of BSA molecules wrapped around the Ag nanoflowers could be observed. The outer BSA layer could act as a multi-functional platform to conjugate targeting antibodies and block the nonspecific adsorption. To further identify proteins are involved into the formation of Ag nanoflowers, FTIR measurements were carried out. As shown in Fig. 3a, the peak at 3420 cm^−1^ was assigned to the combination of the N–H and O–H stretching vibration of BSA. The peak at 2436 cm^−1^ was corresponding to the S–H bond, revealing a slight blue shift owing to the addition of reducing agent and the existence of the auxochromic groups. Two absorption peaks observed at 1650 and 1540 cm^−1^, respectively, represented protein bands for amide I groups at 1610–1690 cm^−1^ consistent with the C=O stretching vibration of peptide linkages and amide II groups due to the C–N stretching and N–H bending. The identified peak at 1398 cm^−1^ was ascribed to the symmetric stretch of COO^−^ from BSA with the carboxyl side groups in the residues of amino acids. The absorption peaks in the region of 1154–1037 cm^−1^ and at 1034 cm^−1^ corresponded to the P–OH and P–O–C stretching, respectively. From the infrared absorption bands, it was confirmed that the primary chemical groups of BSA were reserved.

XRD was further used to analyze the crystalline structure of the Ag nanoflowers. As shown in Fig. 3b, the diffraction peaks corresponded to the (111), (200), (220), and (311) planes. It was possible to index the planes to the face-centered cubic (fcc) structure of Ag. No peaks of any impurities were observed, showing that the products were composed of pure crystalline Ag.

In addition, the MTT cell viability assay (Additional file 3: Figure S3) was conducted to determine the cytotoxicity of Ag@BSA nanoflowers at different concentrations at 37 °C for 24 and 48 h. HSF (1 × 10^4^ cells) were seeded wells in a 96-well plate and then exposed to Ag@BSA nanoflowers. Control experiments were carried out under the same conditions but without the addition of Ag@BSA. When the Ag@BSA concentration was increased from 0.375 to 5 mM at 24 h, no significant viability decreases of HSF were detected in the MTT assay. MTT assays at 48 h (5-mM Ag nanoflowers) showed slightly lower absorbance, but more than 75% of cells were viable, revealing the excellent biocompatibility of Ag@BSA nanoflowers.

### Optimization of Biosensing Conditions

**Fig. 4:**
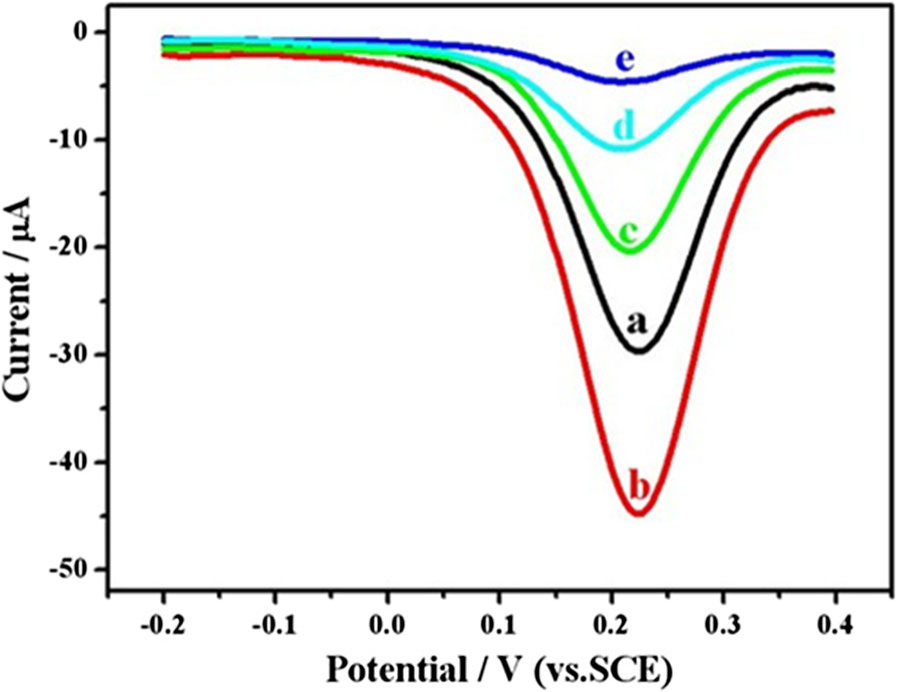
*a* Typical DPV responses of the electrochemically pretreated Au electrode. *b* Formation of the Ag@BSA nanoflower film *c* Conjugation with GA. *d* Immobilization of anti-*E. coli* antibody. *e* Capture of *E. coli* O157:H7 cells (3.0 × 10^3^ cfu mL^−1^), measured in 10 mM, pH 7.4, sterile PBS containing [Fe(CN)_6_]^3−/4−^ (10 mM, 1:1) and 0.1 M KCl

The responses of the [Fe(CN)_6_]^3−/4−^ redox probe reflected the DPV detection sensitivity and was closely associated influenced by various modifications to the immunosensor. Typical DPV measurements at peak current responses on modified electrodes are displayed in Fig. 4 (curves a–e). Compared with the naked Au electrode (curve a), the Ag@BSA nanoflower-modified Au electrode showed a greatly enhanced peak current (curve b), revealing that the Ag nanoflowers could efficiently increase the surface of the electrode and facilitate the electron transfer rate. With further modification of the electrode surface (EDC/NHS, anti-*E. coli*) and incubation (*E. coli*), the peak current gradually decreased (curves c–e). It is noteworthy that the intensity of the peak current was dependent on the numbers of *E. coli* bacteria captured by the antibody-immobilized Ag@BSA nanoflower Au electrode and the amount of anti-*E. coli* required for subsequent immunological identification. In addition, the peak potentials of the modified Au electrodes did not exhibit significant changes, showing that Ag@BSA nanoflowers did not affect the electron transfer kinetics of [Fe(CN)_6_]^3−/4−^.

**Fig. 5:**
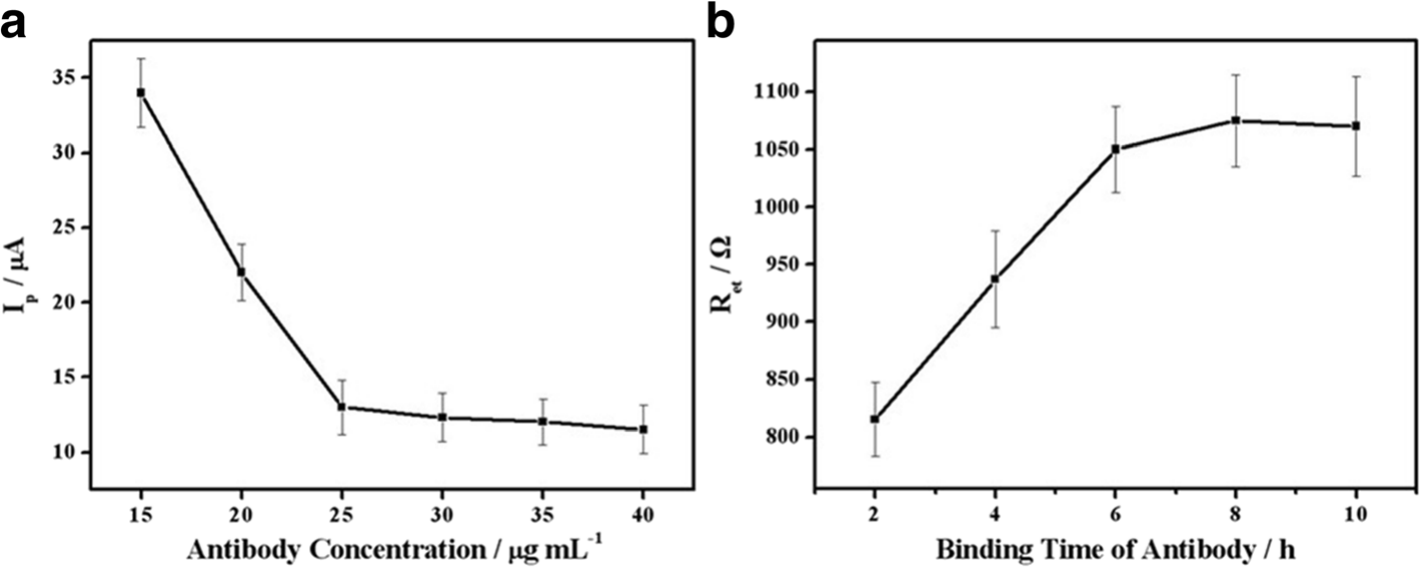
**a** Effects of anti-*E. coli* antibody concentrations on CV responses (scan rate, 100 mV s^−1^). **b** Binding time of anti-*E. coli* antibody on EIS response under different optimal conditions

When fabricating the immunosensor, it was found that the concentration and binding time of antibody were of great importance in determining its analytical performance. With increasing concentrations of anti-*E. coli* in 3 μL volumes, the redox probe peak current was reduced to a minimum value at 25 μg/mL and then marginally increased (Fig. 5a). The results suggested that the total numbers of encapsulated *E. coli* would increase followed by an increased antibody concentration, actions which would significantly influence the redox probe penetration level. Therefore, 25 μg/mL of antibody was chosen as the optimal concentration for subsequent experiments.

The binding time of antibody was also an important parameter, which affected the measurement of *R*_et_. Figure 5b shows the influence of antibody binding time on *R*_et_ differences prior to *E. coli* recognition. It was apparent that the *R*_et_ values exhibited a tendency to increase as a function of an increased antibody binding time, reaching a plateau after 8 h. Thus, 8 h was used as the optimal binding time for anti-*E. coli* to obtain maximal electrochemical signals from the sensor.

### EIS Detection of *E. coli* Based on Anti-*E. coli* Antibody/Ag@BSA Nanoflowers

**Fig. 6:**
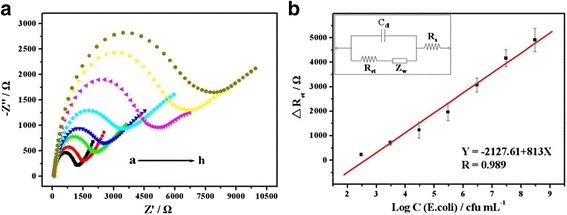
**a** EIS responses of the anti-*E. coli*/EDC/Ag@BSA nanoflower fabricated biosensor at different concentrations of *E. coli* (0, 3.0 × 10^2^, 3.0 × 10^3^, 3.0 × 10^4^, 3.0 × 10^5^, 3.0 × 10^6^, 3.0 × 10^7^, 3.0 × 10^8^ cfu mL^−1^, *a*–*h*) in the presence of a redox probe. **b** Calibration curve for change in electron transfer resistance with logarithm of *E. coli* concentration. *Inset*: the equivalent circuit model

EIS is often used to determine the interfacial properties of electroactive species due to the sensitive and almost instantaneous responses as a result of changes to the surface. Figure 6a was the impedance spectra of the immunosensor that concentrated and captured *E. coli* cells at different initial concentrations. When the bacteria numbers increased, a significant impedance change was recorded. In the range of 3.0 × 10^2^–3.0 × 10^8^ cfu mL^−1^ for bacteria concentration, the increased *R*_et_ value was proportional to the logarithm. The impedance increment was defined as Δ*R*_et_ = *R*_A_ − *R*_B_, where *R*_A_ is the impedance after amplification and *R*_B_ is the impedance after blocking residual nonspecific binding in the absence of *E. coli* incubation. The linear regression equation used was Δ*R*_et_ (Ω) = −2127.61 + 813 lgC_[*E. coli*]_ (cfu mL^−1^) (*R* = 0.989) with a detection limit of 1.0 × 10^2^ cfu mL^−1^ (*S*/*N* = 3) (Fig. 6b).

### Detection Performance of the Immunosensor Stability, Reproducibility, and Specificity

**Fig. 7:**
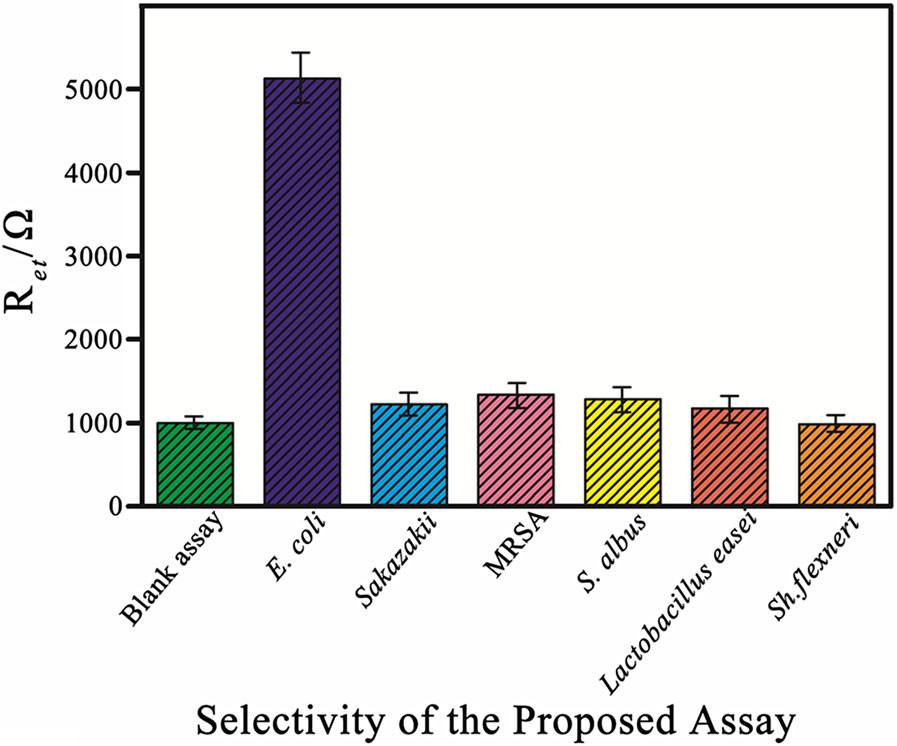
Selectivity of the proposed bacterial sensing strategy. The *error bars* are the standard deviation of three replicate determinations

It was important to evaluate the reproducibility and stability of the immunosensor. Reproducibility was investigated by fabricating various immunosensors under identical optimized conditions. Comparing the *R*_et_^antibody^/*R*_et_^antibody-*E. coli*^ value for different immunosensors, the relative standard deviation (RSD) value of 3% for 20 cfu/mL was obtained (*n* = 3), with excellent reproducibility. The stability of the immunosensors was also investigated by storing them for 30 days in PBS at 4 °C. EIS measurements showed that no significant reduction in their detection capabilities was observed compared to freshly prepared samples. A main focus was to investigate the specificity towards *E. coli*, with *C. sakazakii*, MRSA, *Staphylococcus albus*, *Lactobacillus easei*, and *Shigella flexneri* being used as negative controls. Different types of bacteria at the same concentration (10^6^ cfu/mL) were utilized in the test. After binding with *E. coli*, the electron transfer resistance of the layer-by-layer modified electrode increased by 4000 Ω in contrast to the value obtained in the absence of bacteria. However, a much smaller *R*_et_ increment of 100 and 200 Ω was observed with *C. sakazakii*, MRSA, *S. albus*, *L. easei*, and *Shigella flexneri* captured on the electrode surface, revealing a high specificity of the modified Au electrode sensor immobilized with anti-*E. coli* antibody (Fig. 7).

Table 1 establishes the fact that the sensitivity of the electrode applying Ag@BSA nanoflower as a matrix of immunosensor is comparable to the other recently reported label-free bacterial sensors (references [18–24]). The broad linear range might be attributed to the good conductivity of the inner Ag nanomaterials and the outer BSA molecules hindering nonspecific adsorption.

**Table 1:** Comparison of bacterial detection performance with other reported EIS immunosensors

Used materials	Analyte	Detection range, cfu mL^−1^	Reference
PANI film	*E. coli* O157:H7	1.0 × 10^2^–1.0 × 10^7^	[18]
GO sheets	Sulfate-reducing bacteria	1.8 × 10^2^–1.8 × 10^8^	[19]
GNP-SPCE	Salmonella enteritidis	1.0 × 10^3^–1.0 × 10^5^	[20]
RGSs-CS film	Sulfate-reducing bacteria	1.8 × 10^1^–1.8 × 10^7^	[21]
MA monolayer	*E. coli* O157:H7	3.0 × 10^1^–3.0 × 10^4^	[22]
Nanoporous Al membrane	*E. coli* O157:H7	1.0 × 10^0^–1.0 × 10^4^	[23]
Au-W wires	*E. coli* K12	1.0 × 10^3^–1.0 × 10^8^	[24]
Ag nanoflowers	*E. coli* O157:H7	3.0 × 10^2^–3.0 × 10^8^	This work

*PANI* polyaniline, *GO* graphene oxide, *GNP-SPCE* gold nanoparticle-modified screen-printed carbon electrode, *RGSs-CS* reduced graphene sheets-chitosan, *MA* mercaptohexadecanoic acid

## Conclusions

In summary, we have constructed a Ag@BSA nanoflower electrochemical impedimetric biosensor functionalized with anti-*E. coli* antibody for the rapid, label-free, and specific detection of *E. coli*. The biosensor was capable of detecting very low concentrations of *E. coli* strains with the limit of detection circa 10^2^ cfu mL^−1^ and a broad detection range of 3.0 × 10^2^–3.0 × 10^8^ cfu mL^−1^. We believe that the Ag@BSA electrochemical sensing interfaces will be useful for detecting a wide range of analytes of practical importance including disease-related proteins, cancer cells, and heavy metal ions.

## Additional files

Additional file 1: Figure S1. EDX spectrum of Ag@BSA nanoflowers on Si wafer. (TIF 123 kb)
Additional file 2: Figure S2. SEM images of Ag@BSA microspheres prepared without BSA addition. (TIF 398 kb)
Additional file 3: Figure S3. MTT assays of different concentrations of Ag@BSA nanoflowers to human skin fibroblast (HSF) incubated for 24 h (red column) and 48 h (blue column). (TIF 533 kb)
