# Supplementary figures and images for: Label-Free 3D Ag Nanoflower-Based Electrochemical Immunosensor for the Detection of Escherichia coli O157:H7 Pathogens
Source: Nanoscale Res Lett. 2016 Nov 17;11:507. doi: 10.1186/s11671-016-1711-3 (PMC5114215; doi:10.1186/s11671-016-1711-3)

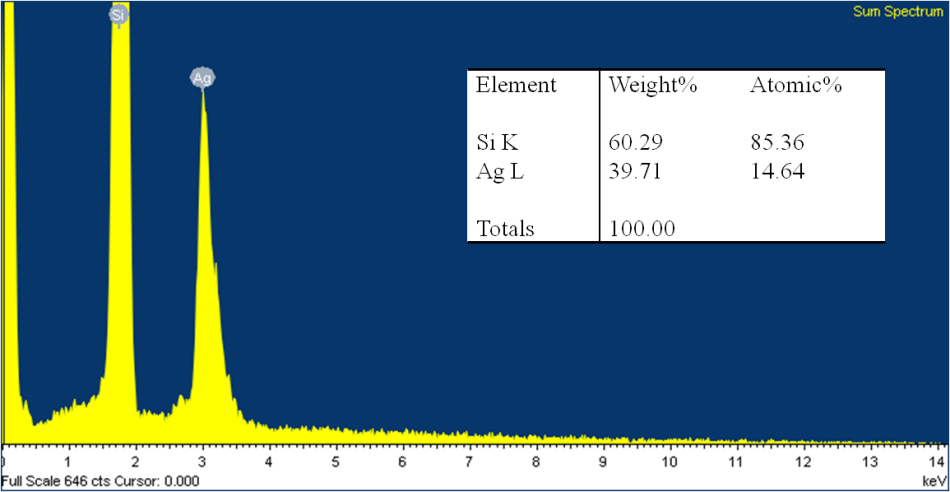

Supplement: Additional file 1: Figure S1. — EDX spectrum of Ag@BSA nanoflowers on Si wafer. (TIF 123 kb) [file 11671_2016_1711_MOESM1_ESM.tif]

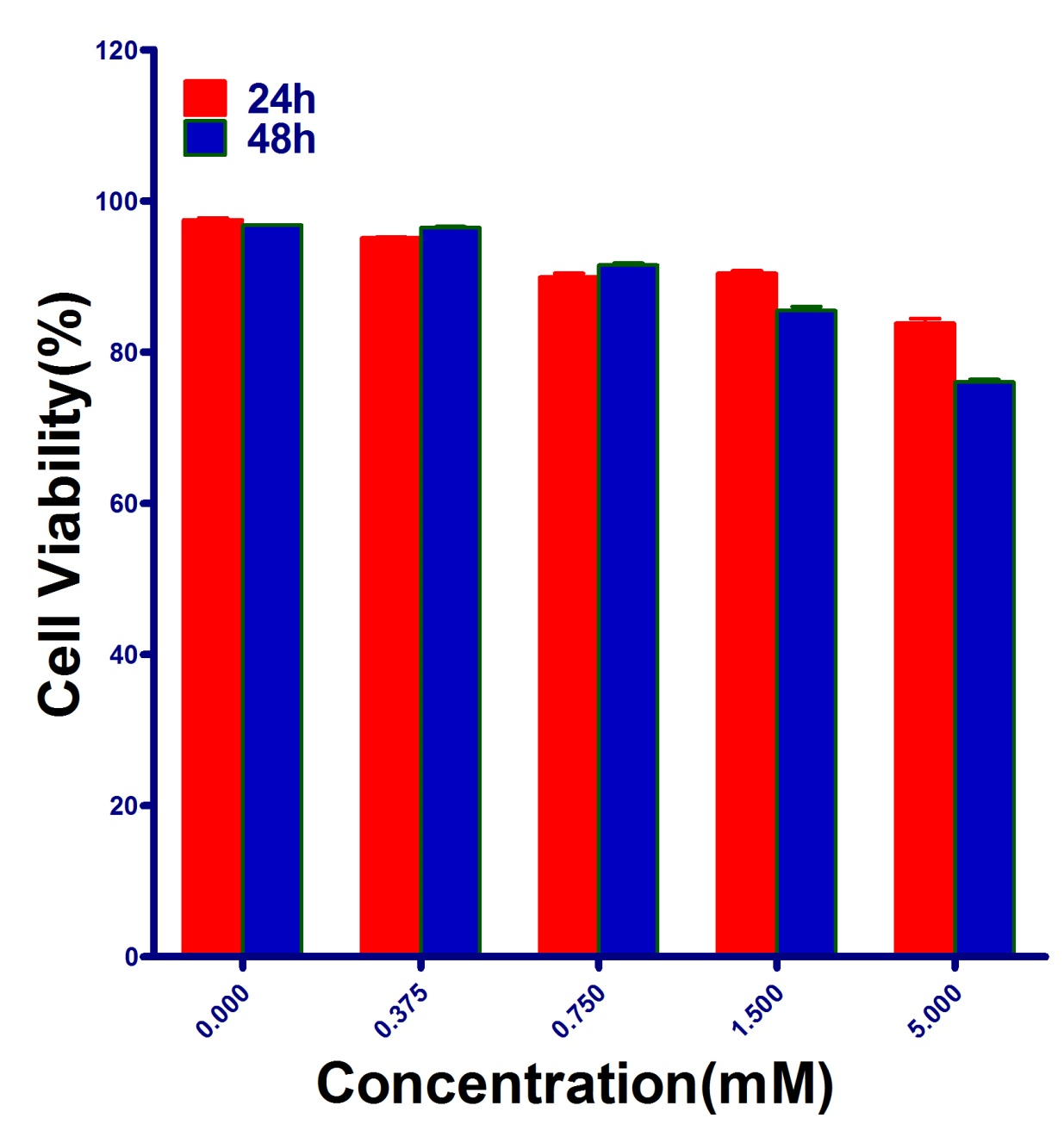

Supplement: Additional file 3: Figure S3. — MTT assays of different concentrations of Ag@BSA nanoflowers to human skin fibroblast (HSF) incubated for 24 h (red column) and 48 h (blue column). (TIF 533 kb) [file 11671_2016_1711_MOESM3_ESM.tif]
